# Detection of splicing events and multiread locations from RNA-seq data based on a geometric-tail (GT) distribution of intron length

**DOI:** 10.1186/1471-2105-12-S5-S2

**Published:** 2011-07-27

**Authors:** Shao-Ke Lou, Jing-Woei Li, Hao Qin, Aldrin Kay-Yuen Yim, Leung-Yau Lo, Bing Ni, Kwong-Sak Leung, Stephen Kwok-Wing Tsui, Ting-Fung Chan

**Affiliations:** 1 School of Life Sciences, The Chinese University of Hong Kong, Shatin, NT, Hong Kong SAR; 2 Department of Computer Science and Engineering, The Chinese University of Hong Kong, Shatin, NT, Hong Kong SAR; 3 School of Biomedical Sciences, The Chinese University of Hong Kong, Shatin, NT, Hong Kong SAR; 4 Hong Kong Bioinformatics Center, The Chinese University of Hong Kong, Shatin, NT, Hong Kong SAR

## Abstract

**Background:**

RNA sequencing (RNA-seq) measures gene expression levels and permits splicing analysis. Many existing aligners are capable of mapping millions of sequencing reads onto a reference genome. For reads that can be mapped to multiple positions along the reference genome (multireads), these aligners may either randomly assign them to a location, or discard them altogether. Either way could bias downstream analyses. Meanwhile, challenges remain in the alignment of reads spanning across splice junctions. Existing splicing-aware aligners that rely on the read-count method in identifying junction sites are inevitably affected by sequencing depths.

**Results:**

The distance between aligned positions of paired-end (PE) reads or two parts of a spliced read is dependent on the experiment protocol and gene structures. We here proposed a new method that employs an empirical geometric-tail (GT) distribution of intron lengths to make a rational choice in multireads selection and splice-sites detection, according to the aligned distances from PE and sliced reads.

**Conclusions:**

GT models that combine sequence similarity from alignment, and together with the probability of length distribution, could accurately determine the location of both multireads and spliced reads.

## Background

Studying gene expressions and understanding how alternative mRNA splicing manifests in a biological system are as essential as elucidation of its underlying regulatory mechanisms [1]. RNA-seq has received much attention as a revolutionary tool for transcriptome analysis [2]. Its massively-paralleled sequencing approach provides paramount advantages over traditional array-based technologies in three key aspects. First, unlike microarrays, RNA-seq has virtually no background signal. It also has no upper limit for transcript-level quantification, which corresponds to the numbers of fragments sequenced. As a result, RNA-seq has a very wide dynamic range compared to microarray. Second, the paired-end (PE) reads – two short sequencing reads separated by a fixed distance - provide information on how two exons are connected. With millions of paired-end reads, analysis of transcript isoforms from a complex transcriptome becomes possible [3]. Finally, RNA-seq does not rely on *a priori* probe information, thereby allowing novel transcripts discovery, cross-validation of gene predictions and genome annotations.

Genomic alignment tools such as BWA [4] and Bowtie [5] can map massive amount of reads onto a reference genome with high efficiency. However, sequences matching multiple locations along the reference genome are handled arbitrarily. Under such circumstances, these ‘multireads’ are randomly assigned to one of the possible locations. Another package, ERANGE, rescues these arbitrarily mapped reads by assigning them in proportion to those uniquely mapped reads [6]. Yet both approaches might distort the abundance of reads that are mapped to paralogous gene families, regions of low sequence complexity or high sequence conservation, thereby affecting virtually all subsequent analysis [7].

Paired-end (PE) information improves alignment precision in genome assembly [8]. The insert size – the separation between two reads in a pair chosen during the sequencing procedures – is an important constraint to help determine the locations of PE reads. The inferred size based on reference mapping should equal to the insert size if no splicing occur within the region that is flanked by two reads within a PE pair. However, such information is of little use when splicing takes place.

Reads spanning two neighbouring transcribed regions cannot be fully mapped onto the reference and are considered as “unmapped” reads. Splicing-aware aligners were thus specifically developed to rescue this unmapped yet expressed, biologically relevant information. Early splicing-aware mapping algorithms started by constructing an artificial splice-junction library using annotated exons, followed by read mapping [6]. Subsequent tools such as G-Mo.R-Se [9] and TopHat [10] perform a global search of splice junctions. However, G-Mo.R-Se is limited by its inability to handle PE reads, whereas TopHat depends on canonical splice codes.

SplitSeek [11], Supersplat [12], and SpliceMap [13] were recently proposed to perform truly global and unbiased spliced-read mapping. These methods, however, rely on an arbitrary read-count method to assess the reliability of a putative splice junction. As a result, the sequencing depth of an experiment has a tremendous effect on splice-site discovery. Recently, Wang et al. proposed a statistical measure of splice sites using a minimum mismatch approach to a database of artificially joined exon-boundary sequences [14]. This approach, however, highly depends on the accuracy of existing exon annotations.

**Figure 1:**
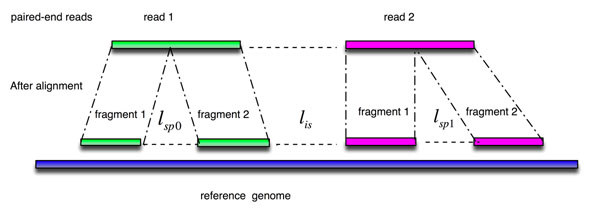
**Alignment of two paired-end (PE) reads.** Read 1 and read 2 could be spliced into two fragments as l_sp0_ and l_sp1_, respectively. l_is_ is the gap length between two PE reads mapped onto the reference genome.

In this study, we have proposed a maximum likelihood estimation (MLE) method based on a geometric-tail (GT) distribution of intron lengths to determine the alignment positions of PE reads. This probabilistic model deals with splice junctions between reads, or those encompassed in one or both of a PE reads (as illustrated in Figure 1). By utilizing *a priori* knowledge, this is a biologically-inspired method to assess the quality of splice junctions. Based on this model, multiple alignments of reads within a PE pair can be properly resolved.

## Results and discussion

### GT distribution

GT distribution has been widely used in the approximation of length distribution for the *de novo* prediction of biological sequence elements, in particular exons and introns. However, fitting geometric distribution of whole-genome intron length has always been suboptimal due to the complex mechanism of gene splicing. There are mainly two types of introns: short ones and long ones, which centered at approximately 100bp and 1000bp, respectively [20]. n-tuple could provide a better fitness using a well-defined n, especially when intron length is small. This allows a better estimation of the tail part by means of a geometric distribution. However, over-fitting would happen when using n-tuple. A smoothing method proposed by Burge [18] could reduce over-fitting. We used an arbitrarily large tuple up to 3,000bp to estimate the distribution of genome-wide intron lengths.

**Figure 2:**
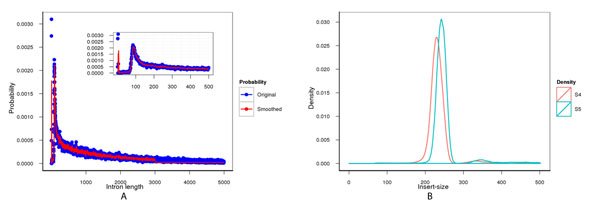
**GT distribution and insert-size distribution**. A) Distribution of intron length and GT estimation. The upper right is an enlarged region from 1 to 500bp. B) Density of insert-size. S4 is marked in red and S5 is marked in green.

As shown in Figure 2A, the blue dots represent different intron lengths, and the red line represents the smoothed probability. Two peaks can be observed around 40bp and 100bp. A large portion of intron lengths span from 500 to 2,000bp, and then declines slightly until 3,000bp.

### Insert-size distribution

Current next-generation sequencing platforms such as Roche 454, ABI SOLiD, and Illumina Genome Analyzer all employ PE sequencing techniques. Fragments with a defined size were isolated and subjected to downstream sequencing steps. A rough estimation of the insert size was usually given during the sample preparation steps. A more precise measure could only be inferred from mapping results.

We adopted the concept of “regular pair” defined in BWA, which has extreme values filtered from each 256*1024 pairs. We used the uniquely mapped PE pairs to estimate the distribution (Figure 2B). Insert-size distributions in the two datasets S4 and S5 approximated a normal distribution, and they were used to calculate the convolution probabilities.

### Multiread analysis

Non-uniquely mapped reads (multireads) refer to those matching multiple locations along the reference genome. BWA uses a random selection method to tackle multireads, which might introduce errors. Multireads can be classified into three groups according to their characteristics: 1) expressed repetitive elements, 2) transcripts from distinct loci that share great similarity with other counterparts, and 3) one or both reads from a PE pair derived from splicing events, and yet happened to have a full match found on the reference. Group 1 can easily be handled according to its high occurrences and annotations, while Groups 2 and 3 are difficult to resolve simply by alignment. The GT-based estimation we have proposed here could help mitigate such selection. We took Groups 2 and 3 into account by running ABMapper [21] on repetitive reads that could be mapped for more than 300 times onto the genome. When tracing back these reads to the mapping result by BWA, we found all these reads were tagged with “XT:A:R”, and BWA randomly selected the locations of these multireads.

GT-based models shared at least 88% of the mapping locations with BWA. The two sets differed by 6-10%. Inconsistency between the two sets of results was subsequently evaluated based on gene expression levels. Expression level of a given gene was calculated using a normalized read-count method, which involves summing the total number of reads mapped to the gene followed by normalization with the gene length. Based on the assumption that a multiread is more likely to be originated from a highly expressed region than from a lower one, we consistently observed more than 60% of the reads in the ‘different’ groups in all three of our GT-based models that showed higher expression levels than those from BWA (Additional file 1, Table 1B). These results have demonstrated that the GT-based model is likely to be more accurate than BWA in the selection of multireads position.

### Splice-site comparison

**Figure 3:**
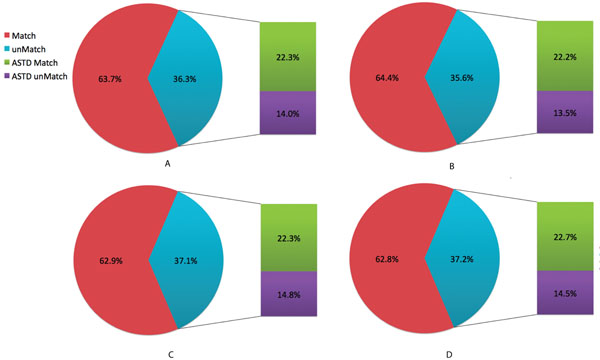
**A comparison of splice junctions between the GT-based model, Tophat, and SpliceMap, and with the ASTD**. A) S4 GT-based model with SpliceMap; B) S5 GT-based model with SpliceMap; C) S4 GT-based model with TopHat; and D) S5 GT-based model with TopHat.

SpliceMap and TopHat were used to evaluate the accuracy of GT-based models in splice-site detection. We did not use SplitSeek in our comparison because it only supports SOLiD data. SpliceMap and TopHat were applied to the same datasets (S4 and S5) and the results were compared to the Alternative Splicing and Transcript Diversity (ASTD) database [16]. The ASTD database is the largest depository of experimentally-verified splicing events. Junction sites from the GT-based models were compared to those identified by TopHat and SpliceMap. We defined that two putative splice sites, one identified by the GT-based model and the other identified either by TopHat or SpliceMap, are a match if positions of the two are within 8-bp from each other. The GT-based models, TopHat, and SplicMap shared a large number of common splice junctions (Figure 3). The unmatched sites between the GT-based models and Tophat or SpliceMap were then further compared with the ASTD entries using the same criteria.

In all three conditions, GT-based models clearly exceled in the detection of putative splice junctions: with 53% and 49% more reported sites compared to SpliceMap and TopHat, respectively (Table 1). As shown in Figure 3 (detailed numbers in Additional file 1, Table 2), over 60% of unmatched junctions could be confirmed by the ASTD. These ASTD-confirmed junctions constituted approximately 22% of the total predicted splice junctions based on the GT-model. These results indicated that both TopHat and SpliceMap have missed at least one-fifth of true splice junctions.

**Table 1:** Splice sites comparison and EST validation

S4	Model 1	Model 2	Model 3
TOTAL splice sites	102074	102784	101974
Confirmed by EST	98109	98819	98008
% confirmed by EST	96.12%	96.14%	96.11%
Splice sites by TopHat	66988		
Splice sites by SpliceMap	66452		

**S5**	**Model 1**	**Model 2**	**Model 3**

TOTAL splice sites	89249	90269	89189
Confirmed by EST	85938	86958	85878
% confirmed by EST	96.29%	96.33%	96.29%
Splice sites by TopHat	60659		
Splice sites by SpliceMap	60625		

Some splice junctions reported by our GT-based model, TopHat and SpliceMap were not in the ASTD, either because the splice sites were not deposited in the ASTD, or the splice sites were false positives. We therefore performed an exhaustive search on all splice junctions reported by the GT-based model against the Human EST database. We found that the GT-based model achieved a remarkable 96% accuracy in finding true splice junctions (Table 1). Therefore, a majority of the splice junctions reported by the GT-based model and were unmatched to the ASTD were indeed true splice sites.

## Conclusions

RNA-seq has advanced the field of biological research. It has increasingly been used for transcriptomic analysis of model organisms as well as disease models in human. For non-model organisms, however, since gene annotation is often incomplete or completely absent, there is insufficient data for GT distribution estimation. In such cases, GT distribution will have to be estimated with an evolutionary-related species.

In this study we have shown that the GT-based model, which employs an empirical distribution of intron lengths, has an edge over the current mainstream methods such as TopHat and SpliceMap. Two main reasons behind this advantage are (1) ABMapper, the spliced-aware mapper that has much higher sensitivity and identify far more putative splice sites than other tools [21]; and (2) the GT-based model itself, which is capable of multireads mapping and splice-site inference. We have shown that combing these two factors would lead to the discovery of 50% more EST-validated splice sites than existing tools.

## Methods

### Workflow

Two human lymphoblastoid cell-line datasets (identified as S4 and S5 throughout this study) were used in testing our model. PolyA-selected mRNAs were sequenced by the Illumina platform at the Beijing Genomic Institute-Shenzhen. S4 and S5 were sequenced with 75 base pair (bp) in read length and with an approximately 250bp insert size. The two datasets comprise of 4.6 million and 3.8 million of PE reads, respectively. Reads were trimmed to 67 bp to get rid of low quality bases. Ensembl transcripts and gene annotations were retrieved from the UCSC genome browser [15]. Splicing events and junction-site information were retrieved from the Alternative Splicing and Transcript Diversity (ASTD) database [16]. Human genome version hg18 was used as the reference for read mapping. Human expressed sequence tags (ESTs) were retrieved from NCBI as of July 2, 2010.

The internal distance between two adjacent exons was calculated based on the Ensembl gene annotations. A geometric-tail (GT) distribution of internal distances was calculated as described in the Algorithm section. We used a Gaussian smoothing method proposed by C. Burge to replace each point with a normally-distributed variable centered at the point to avoid over-fitting [17]. The distribution of gap lengths between mapped PE reads was calculated by a convolution of the normal distribution of insert-sizes and the GT distribution of intron lengths.

Reads were mapped to the hg18 reference genome using ABMapper, which was specifically developed by our group for multiread and spliced alignments [21]. Internal testing has shown that it outperforms current splicing-aware aligners in splice-site detection. All putative locations of aligned reads were kept for probability calculation after repetitive reads filtering, which involves removing reads that occur more than 300 times in the genome. A maximum likelihood method was used to generate three models and were subsequently used to calculate the probability (detailed in the Algorithm section).

**Figure 4:**
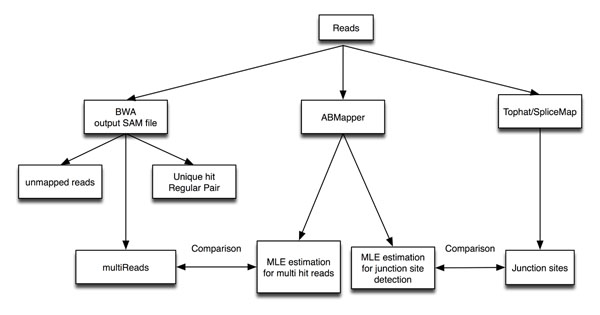
**Workflow of this study.** Comparison of the GT-model to TopHat and SpliceMap

BWA output that contains randomly selected multireads was extracted for comparison to our GT-model. Reads were first mapped by BWA (v0.4.9) onto hg18. Multireads with the tag “XT:A:R” were then extracted for downstream analysis (as shown in Figure 4). SpliceMap (v3.1.1) and TopHat (v1.0.14) were used to perform splice-site detection. ELAND was used as the default aligner in SpliceMap; and Bowtie (v0.12.5) was used with TopHat.

### Algorithm

Our empirical probabilistic model of intron length utilizes a maximum likelihood estimation method to determine the most probable location for multireads and to detect splice junctions.

Length-distribution estimation has been widely used on many biological elements. For example, C. Burge used a shifted-geometric distribution to estimate the length of introns in a gene finding algorithm [17,18]. However, the distribution of intron length is far more complex than other biological sequence elements because the splicing mechanism is complicated and is not yet fully understood. A combination of more than one distribution is required to properly describe it. Here we have adopted a geometric-tail (GT) distribution to represent the intron sequence length, which was first proposed in [19].

GT distribution is a two-part distribution: the first is an arbitrary length distribution, and the second is a geometric distribution. The definition is as follows:

where *d_x_* is defined by a t-tuple (*d*_1_, *d*_2_, …, *d_t_*_– 1_), and parameter q (0≤q≤1) satisfies

The parameters could be estimated as [19]:

where γ(i) is the number of times that length i occurs

To avoid over-fitting of *d_x_*, we used a Gaussian smoothing procedure as previously described by others [18].

Mapping of two reads in a pair onto the reference genome could result in two types of gaps (as illustrated in Figure 4): (1) *l_is_*: gaps that are flanked by the PE reads (‘read 1’ and ‘read 2’) when they are mapped onto the reference genome; and (2) *l_sp_* : spliced gaps at variable lengths that are introduced when a single read is split into two fragments (‘fragment 1’ and ‘fragment 2’) during the read-mapping process . *l_sp_* is the gap between two fragments introduced mainly by splicing. Hence the sequence between the two mapped fragments is therefore considered an "intron".

All the putative locations would form a set of gap lengths *L*{*l_sp_***_0_**, *l_sp_***_1_**, …, *l_spn_*, *l_is_*}, which includes intron sequence lengths {*l*_*sp*0_, *l*_*sp*1_, …, *l*_*spn*_} and insert gaps {*l_is_*}, with the corresponding probability *P_l_*{*P_sp_***_0_**, *P_sp_***_1_**, …, *P_spn_*, *P_is_*}. The length of spliced gap (*l_sp_*) would follow the empirical GT distribution for it actually represents the intron length between two fragments of a reads *P*(*X* = *l_sp_*) = *δ*(*l_sp_*). However, the distribution of insert gap (*l_is_*) is more complex than that of intron sequence length (*l_sp_*), because it is a combination of spliced-gap distribution and insert-size distribution introduced during paired-end sequencing. Spliced-gap distribution is an empirical GT distribution, and the distribution of insert-size is considered as a normal distribution according to the amplicon selection procedure (defined during the actual RNA-seq procedures). Let *d_i_* be the insert size of a PE read in the whole dataset, and I be the length of the insert fragment library in the experiment, then:

*d_i_* = *g*(*x*) ~ *N*(*I*, *σ*^2^).

So the distribution of *l_sp_* is a convolution of GT distribution and the normal distribution:

*P*(*X* = *l_is_*) = (*g* * *δ*)(*l_is_*)

The likelihood function for each putative location θ of a PE read is:

The maximum likelihood is estimated to find the most probable location for a pair of PE reads. Besides length distribution, expression levels and splice-site frequency are also used as *a priori* knowledge to help determine read locations. The expression level is calculated by the summation of all expressions on each mapped position along the genome, which is normalized by the total length of mapped reads. The splice-site probability is similar to the read-count method, and total count of splice sites is used for normalization. The following are the three models used in our study:

‘GT model 1’: MLE estimation WITHOUT any *a priori* knowledge;

‘GT model 2’: MLE estimation with expression level as *a priori* knowledge;

‘GT model 3’: MLE estimation with junction-site frequency as *a priori* knowledge.

## Competing interests

The authors declare that they have no competing interests.

## Authors' contributions

SKL carried out the programming and drafted the manuscript. JWL performed the wet-lab experiment. SKL, JWL, HQ, and AKY performed data analysis. LYL and BN refined the model and assisted in the coding with SKL. KSL, KWT and TFC supervised the project. TFC revised the manuscript. All authors have contributed, read, and approved the final manuscript.

## Supplementary Material

Additional file 1 Click here for file
