# Supplementary material for: Detection of splicing events and multiread locations from RNA-seq data based on a geometric-tail (GT) distribution of intron length
Source: BMC Bioinformatics. 2011 Jul 27;12(Suppl 5):S2. doi: 10.1186/1471-2105-12-S5-S2 (PMC3226252; doi:10.1186/1471-2105-12-S5-S2)
Supplement: Additional file 1 [file 1471-2105-12-S5-S2-S1.docx]

## Additional file 1

## Table 1

**(A)**

**A comparison of multiread locations from BWA alignment and GT-based model estimation**

| Compared to BWA | S4 | | | S5 | | |
| --- | --- | --- | --- | --- | --- | --- |
|  | Model 1 | Model 2 | Model 3 | Model 1 | Model 2 | Model 3 |
| Same | 873140(95%) | 857521(93%) | 858844(94%) | 730026(92%) | 698961(88%) | 701876(89%) |
| Different | 43955(5%) | 59574(7%) | 58252(6%) | 63268(8%) | 94333(12%) | 91418(13%) |
| Total | 917105 | 917105 | 917105 | 793294 | 793294 | 793294 |

**(B)**

**A comparison of gene expression levels derived from BWA and the GT-based model**

| Datasets | GT models | GT model higher | BWA higher | Same expression | GT model unknown | BWA  unknown | Both unknown |
| --- | --- | --- | --- | --- | --- | --- | --- |
| S4 | Model 1 | 17330 | 6636 | 2678 | 5652 | 8861 | 2799 |
|  | Model 2 | 21453 | 10422 | 3329 | 9644 | 10561 | 4166 |
|  | Model 3 | 21518 | 9851 | 3239 | 9441 | 10275 | 3928 |
| S5 | Model 1 | 33352 | 12165 | 2935 | 2619 | 10064 | 2134 |
|  | Model 2 | 38125 | 21145 | 3347 | 13513 | 14159 | 4045 |
|  | Model 3 | 36533 | 20913 | 3262 | 13412 | 13544 | 3755 |

## Table 2

**Junction sites comparison of the GT model to TopHat and SpliceMap**

|  | S4 vs SpliceMap | | | | S5 vs SpliceMap | | | |
| --- | --- | --- | --- | --- | --- | --- | --- | --- |
|  | Model1 | Model2 | Model3 | Average % | Model1 | Model2 | Model3 | Average % |
| Matched | 64938 | 64891 | 64970 | 63.7% | 57639 | 57656 | 57644 | 64.4% |
| Unmatched |  | | | | | | | |
| ASTD matched | 22752 | 22788 | 22698 | 22.3% | 19848 | 19916 | 19825 | 22.2% |
| ASTD unmatched | 14384 | 14105 | 14306 | 14.0% | 11762 | 12697 | 11720 | 13.5% |
|  |  |  |  | 100% |  |  |  | 100.0% |

|  | S4 vs TopHat | | | | S5 vs TopHat | | | |
| --- | --- | --- | --- | --- | --- | --- | --- | --- |
|  | Model1 | Model2 | Model3 | Average % | Model1 | Model2 | Model3 | Average % |
| Matched | 64373 | 64233 | 64331 | 62.9% | 56295 | 56205 | 56251 | 62.8% |
| Unmatched |  |  |  |  |  |  |  |  |
| ASTD matched | 22778 | 22824 | 22749 | 22.3% | 20297 | 20408 | 20297 | 22.7% |
| ASTD unmatched | 14923 | 15727 | 14894 | 14.8% | 12657 | 13656 | 12641 | 14.5% |
|  |  |  |  | 100% |  |  |  | 100% |
